# Bilateral Vestibulopathy in Superficial Siderosis

**DOI:** 10.3389/fneur.2018.00422

**Published:** 2018-06-06

**Authors:** Sang-Yeon Lee, Dong-Han Lee, Yun Jung Bae, Jae-Jin Song, Ji Soo Kim, Ja-Won Koo

**Affiliations:** ^1^Department of Otorhinolaryngology, Seoul National University College of Medicine, Seoul National University Bundang Hospital, Seongnam, South Korea; ^2^Department of Radiology, Seoul National University College of Medicine, Seoul National University Bundang Hospital, Seongnam, South Korea; ^3^Department of Neurology, Seoul National University College of Medicine, Seoul National University Bundang Hospital, Seongnam, South Korea

**Keywords:** superficial siderosis, vertigo, hearing loss, cerebellar ataxia, vestibulopathy

## Abstract

**Background:** Superficial siderosis (SS) is a rare condition in which hemosiderin, an iron storage complex, is deposited in neural tissues because of recurrent subarachnoid bleeding. Hemosiderin deposition in the vestibulocochlear nerve (CN VIII), brain, spinal cord and peripheral nerve can cause sensorineural hearing loss (SNHL) and postural imbalance, but much remains unknown about the vestibular manifestations of SS.

**Objectives:** To report the clinical course, cochleovestibular status, and patterns of vestibulopathy during follow-up of a relatively large case series, and to discuss the possible pathophysiological mechanism of vestibular deterioration.

**Methods:** Six patients diagnosed with SS by magnetic resonance imaging (MRI) were enrolled. Their medical records and radiological findings were retrospectively reviewed, particularly in terms of progression of the vestibulocochlear manifestations and the radiological characteristics.

**Results:** All six patients had SNHL. Five of them exhibited progressive hearing loss over years, which was asymmetric in four. On their most recent evaluations, patients showed cerebellar ataxia with combined central and peripheral vestibulopathy on both sides (*n* = 4), a bilateral peripheral vestibulopathy (*n* = 1) or isolated central vestibulopathy (*n* = 1). Notably, the former four patients showed an evolution of isolated central vestibulopathy into combined central and peripheral vestibulopathy. Hypo-intense lesions on T2 weighted MRIs were evident around the cerebellum in all patients, but such lesions were observed around the brainstem in five and the CN VIII in four. The cochlea-vestibular dysfunction generally progressed asymmetrically, but no left-right asymmetry was evident on MRI.

**Conclusions:** SS typically presents as bilaterally asymmetric, progressive cochleovestibular dysfunction with cerebellar ataxia. The pattern of vestibular dysfunction is usually combined central and peripheral vestibulopathy on both sides. Thus, precise identification of audiovestibular dysfunction and central signs is essential in SS, and patients with SS should undergo regular, comprehensive neurotological evaluation to optimize their treatments and prognosis.

## Introduction

Superficial siderosis (SS) is a rare condition in which hemosiderin, an iron-storage complex, is deposited in neural tissues because of recurrent subarachnoid bleeding (1). SS may be considered as a central nervous system (CNS) disease that clinically manifests as cerebellar ataxia, pyramidal signs, and dementia (2). However, the symptoms can vary depending on the distribution of hemosiderin deposition; deposition in the cerebellum and vestibulocochlear nerve (CN VIII) can cause sensorineural hearing loss (SNHL) in addition to cerebellar ataxia and postural imbalance (3, 4). Furthermore, patients with SS mostly experience deterioration of vestibular function on both sides (5, 6). A recent case series showed that chronic bilateral central vestibulopathy coexisted with peripheral vestibulopathy, especially when hearing impairment was evident (7). However, another study reported that only bilateral peripheral vestibulopathy is evident in SS patients (8). Such inconsistent results suggest that misidentification of vestibular status in SS patients may pose diagnostic and therapeutic challenges, especially during rehabilitation therapy employing the vestibulo-ocular reflex (VOR) in which identification of the precise vestibular status is of critical importance (5, 9).

Although hemosiderin deposition in the CNS and around CN VIII is associated with vestibular manifestations, most publications have focused on audiological features including hearing deterioration (10). To date, vestibular deficits have been reported in less than 30 patients with SS, mostly without follow-ups for vestibular function (3, 5–9, 11–22). To the best our knowledge, not much attention has been paid to the evolution of vestibular function and its pathophysiological mechanisms in patients with SS.

Herein, we explore the progression of balance and hearing function, and patterns of vestibulopathy during follow-up of six patients. We also suggest a possible pathophysiological mechanism for the evolution of vestibular features.

## Materials and methods

### Subjects

We retrospectively reviewed the charts of eight patients diagnosed with SS in Seoul National University Bundang Hospital between 2005 and 2016. Of these, long-term systematic neurotological evaluations were scheduled for six patients (the subjects of the present study). One included patient (subject 2) was previously described in a case report that we authored (23). This study was approved by the Seoul National University Bundang Hospital Institutional Review Board (no. IRB-B-1710-427-106) and was conducted in accordance with all relevant tenets of the Declaration of Helsinki.

### Neurotologic evaluation

Pure tone audiometry (PTA) and speech audiometry (SA) were performed. Hearing thresholds were calculated by averaging the PTA thresholds at 0.5, 1, 2, and 3 kHz based on the American Academy of Otolaryngology-Head and Neck Surgery (AAO-HNS) guidelines. Progression of hearing loss was defined as declines in the audiometric thresholds >10 dB HL at three frequencies, ≥15 dB HL at two frequencies, and/or ≥20 dB HL at one frequency, over the follow-up period. The hearing thresholds for seven different frequencies (0.25, 0.5, 1, 2, 3, 4, and 8 kHz) were evaluated in a soundproof booth, and the audiometric configuration of each subject categorized as flat (the thresholds across the tested frequencies did not vary by >20 dB HL); high tone hearing loss (equal or successively increasing thresholds from 0.25 to 8 kHz and the difference between the thresholds at 250 and 8,000 Hz was >20 dB HL); and low tone hearing loss (equal or successively decreasing thresholds from 0.25 to 8 kHz and the differences between the thresholds at 250 and 8,000 Hz were >20 dB HL) (24).

Eye movements were assessed using a video-oculography (VOG) system or a videonystagmography (VNG) system (SMI, Teltow, Germany; or ICS Medical, Schaumburg, IL, USA) with patients in the sitting position during both spontaneous and induced nystagmus. Spontaneous nystagmus (SN) was analyzed both with and without fixation; all subjects attempted to look straight ahead. Gaze-evoked nystagmus (GEN) was also evaluated. Induced nystagmus was evaluated during positioning, head-shaking, and when vibration was applied to each side of the mastoid tip for 10 s with the aid of a VVIB 100 device (Synapsis, Marseille, France). Head-shaking nystagmus (HSN) was assessed 15 s after passive head-shaking with the neck flexed by 30° at a frequency of ≥2 Hz.

The bithermal caloric test was performed with water caloric stimulator NCI480 (ICS medical, Schaumburg, IL, USA) in the supine position with head elevation at 30°C. Caloric irrigation was delivered in the order of right cool (30°C), left cool (30°C), right warm (44°C), and then left warm (44°C) for 30 s with a flow rate of 300 ml/min. The maximum slow-phase velocity (SPV) of nystagmus was calculated after irrigation at each temperature, and canal paresis (CP) was determined using Jongkees' formula (25). If nystagmus was not induced during caloric stimulation, ice water test was conducted by using 40 ml of ice water (4°C) irrigation for 30 s in the supine position and then in the prone position to see if the direction of induced nystagmus changes (26).

The rotator chair test was performed in the earth vertical axis rotation unit (CHARTR RVT system, ICS Medical). The subject's head was positioned and restrained on the head rest with neck flexion by 30°C. Horizontal VOR was recorded with an electronystagmography system. Rotational stimulus was sinusoidal harmonic acceleration (SHA), and impulse acceleration and deceleration (step velocity). On SHA test, peak velocity was 60°C per second and rotation frequencies were 0.01, 0.02, 0.04, 0.08, 0.16, 0.32, and 0.64 Hz. Parameters of SHA test included gain, phase, and symmetry (27). Test protocol of the step velocity stimulation was angular acceleration of 100°C per second for 1 s, rotation at a constant velocity (100°C per second) for 60 s, and then deceleration to 0 degree per second within 1 s. Parameter for rotational test was time constant of nystagmus diminution after impulse acceleration and deceleration. The time constant after impulse acceleration toward the lesion side and that after impulse deceleration toward the healthy side were averaged (ipsilesional time constant, Tci), and the time constant after impulse acceleration toward the healthy side and that after impulse deceleration toward the lesion side were averaged (contralesional time constant, Tcc). Normal value of Tc (mean T ± 2SD) obtained from this unit ranged from 11 to 21 s (28).

Head impulse test (HIT) was performed using a video HIT system for acquisition and analysis of the eyeball and head movements (SLMED, Seoul, Korea). The examinees were instructed to stare at a stationary target at a distance of 1 m in front of them while short lasting head rotations around an earth-vertical axis were randomly applied from behind the examinees. The test was repeated at least 10 times on each side in an unpredictable direction with 5–10^o^ and peak accelerations of 750–6000^o^/sec (29). Only head rotations with a defined waveform within a predefined velocity and acceleration window were accepted. The movements of the right eyeball and the head were recorded. The area under the velocity curves of these two movements was obtained from head-impulse onset to the back crossing of zero. VOR gain on video HIT was defined as the ratio of the area under the velocity curves of the right eye to that of the head (30). The VOR gains were measured for individual trials as the ratio of the mean eye velocity divided by the mean head velocity during a 40-ms window centered at the time of peak head acceleration (31). We defined abnormal HIT findings when the mean VOR gain was less than the mean minus 2 SDs of the control data (i.e., <0.88 for the HC, <0.75 for the AC, and <0.77 for the PC).

Cervical vestibular-evoked myogenic potentials (cVEMP) were recorded with the subject supine on a bed with the head raised ~30° from the horizontal and rotated contralaterally in order to activate the sternocleidomastoid (SCM) muscles. The surface EMG activity was measured from an active electrode placed over the belly of the contracted SCM after subtracting activity from a reference electrode located on medial clavicle. A ground electrode was attached to the forehead. cVEMP were recorded using a Nicolet Viking Select unit (Nicolet-Biomedical, Madison, WI, USA). A short burst of alternating tone (110 dB nHL, 123.5 dB SPL, 500 Hz, rise time = 2 ms, plateau = 3 ms, fall time = 2 ms) was applied at 2.1 Hz monaurally via a headphone. The analysis time for each stimulus was 50 ms and responses elicited by up to 80 stimuli were averaged for each test. The signal was bandpass filtered at 30–1,500 Hz, and the mean values of at least two trials were obtained from each ear for all participants. During each recording, the amplified EMG activities of the SCM were also monitored and digitized at 1 kHz using an analog-todigital converter (NI PCI-4461, National Instruments, Austin, TX, USA). The LabVIEW program (National Instruments, Austin, Texas, USA) was used to analyze the peak to peak amplitudes and calculate the mean tonic activation during the recording. The absolute cVEMP amplitude was then normalized against the mean tonic activation of the SCM during the recording. To compare the normalized p1 – n1 amplitudes of the cVEMP between the sides, the interaural difference ratio of the normalized amplitudes (IAD, %) was also calculated as [(AR – AL) / (AR + AL) × 100], where AR and AL are the normalized p1-n1 amplitude on the right and left sides, respectively. Both the p1 and n1 peak latencies were also calculated (32). In this study, we defined normal range of cVEMP when the IAD ratio was less than 22.5%.

Unilateral vestibular hypofunction (UVH) was diagnosed if catch-up saccades in a single direction were evident on HIT; and if the canal paresis was >25% or the sum of the maximum SPV on a single side (R44+R30 or L44+L30) was <10°/s. Bilateral vestibular hypofunction (BVH) was diagnosed when the sum of the maximum SPVs under four conditions (R44+R30+L44+L30) was <12°/s, or if no VOR was evident on the rotation chair test.

### Radiologic evaluation

Magnetic resonance imaging (MRI) was performed using a 3-T MRI scanner (Achieva and Ingenia; Philips, Best, the Netherlands) with a 32-channel SENSE head coil (Philips Healthcare). All subjects underwent brain MRI with T2-weighted imaging (TR, 3,000 ms; TE, 80 ms; FOV, 185 × 230 mm^2^; acquisition matrix, 420 × 375; slice thickness, 5 mm; slice gap, 1 mm; flip angle, 90°) and/or T2^*^-gradient recalled-echo (GRE) imaging (TR, 800 ms; TE, 18 ms; FOV, 185 × 230 mm^2^; acquisition matrix, 256 × 256; slice thickness, 5 mm; slice gap, 1 mm; flip angle, 23°). Thin-section internal auditory canal imaging was additionally performed in 4 subjects using T2-weighted volume isotropic turbo spin-echo acquisition (VISTA) (TR, 2,000 ms; TE, 250 ms; FOV, 160 × 160 mm^2^; acquisition matrix, 228 × 228; slice thickness, 0.7 mm; overlapping, 0.35 mm; flip angle, 90°) and balanced turbo field-echo (bTFE) (TR, 8.5 ms; TE, 4.3 ms; FOV, 150 × 150 mm^2^; acquisition matrix, 224 × 336; slice thickness, 1.4 mm; overlapping, 0.7 mm; flip angle, 50°) sequence. A neuroradiologist blinded to the clinical information assessed the extent and the location of hemosiderin deposits including cerebellum, brainstem, and CN VIII.

## Results

### Case reviews

The clinical characteristics, clinical courses, neurotological evaluations, and laboratory data of our six SS subjects are summarized in Tables 1–4.

**Table 1:** Clinical characteristics and clinical course in our subjects with superficial siderosis.

**Subject**	**Sex**	**Age**	**Etiology of SS (onset, ago)**	**Initial neuro-otologic symptoms**	**Duration: From event to initial symptoms**	**Sequential symptoms (Time duration from initial symptom)**	**Audiologic manifestations**	
							**Characteristics**	**Duration of progression**
1	F	78	Idiopathic	CA	-	B) HL (4years) Oscillopsia (9years)	Non-progressive symmetric SNHL	Not affected
2	M	38	Head trauma (14 years ago)	B) HL, disequilibrium	2 years	Hyposmia (1year)	Progressive asymmetric SNHL	1 year
3	F	42	Brain surgery due to chordoma (18 years ago)	CA	10 years	B) HL (3 years) B) dysesthesia (5 years)	Progressive asymmetric SNHL	8 months
4	F	52	Brain hemorrhage due to cavernous hemangioma (20 years ago)	L)HL	8 years	CA, disequilibrium (1 year)	Progressive asymmetric SNHL	5 years
5	F	53	Subarachnoid bleeding in sacrum level (6 years ago)	CA, B) HL	2 years	Diplopia (3 months)	Progressive symmetric SNHL	2 years
6	M	65	CNS surgery due to lumbar cystic tumor (20 years ago)	CA	10 years	R) HL (7 years)	Progressive asymmetric SNHL	1 year

*M, male; F, female; B, bilateral; R, right; L, left; CA, cerebellar ataxia; HL, hearing loss; SNHL, sensorineural hearing loss*.

**Table 2:** Neurotologic evaluations in our subjects with superficial siderosis^†^.

	**Subject 1**	**Subject 2**	**Subject 3**	**Subject 4**	**Subject 5**	**Subject 6**
**VIDEO NYSTAGMOGRAPHY**						
Spontaneous	DB, RB	–	subtle DB	–	–	subtle DB
Gaze-evoked	DB	–	DB, RB/ DB, LB	–	RB/LB	–
Vibration	–	–	DB, RB	LB	LB	DB, RB
Head shaking	DB, RB	–	DB	LB	-	DB
Head thrust	–	BCU	BCU	-	BCU	BCU
**OCULAR MOTOR TEST**						
Pursuit gain	BD	Normal	BD	BD	BD	Normal
Saccade	Hypometria	Normal	Hypermetria	Normal	Hypometria	Hypermetria

† *If positive signs of each variables were identified at least once during several neurotologic evaluations, we documented the positive findings in Table 2. DB, down beating; RB, right beating; LB, left beating; BCU, bilateral catch up saccade; BD, bilaterally decreased*.

**Table 3:** Laboratory evaluations in our subjects with superficial siderosis.

	**Subject 1**	**Subject 2**	**Subject 3**	**Subject 4**	**Subject 5**	**Subject 6**
**CALORIC TEST (INITIAL)**						
SPV(RW+RC), deg/sec	37	4	1	32	9	38
SPV(LW+LC), deg/sec	34	0	1	31	0	36
Ice water test		NR	NR		NR	
**CALORIC TEST (LAST F/U)**						
SPV(RW+RC), deg/sec	4			2		N/A
SPV(LW+LC), deg/sec	4			0		N/A
Ice water test	NR			NR		N/A
**ROTATOR CHAIR TEST (LAST F/U)**						
Gain	Decrease	Decrease	Decrease	Decrease	Decrease	N/A
Phase	Lead	Lead	Lead	Lead	Lead	N/A
Symmetry	Symmetry	Symmetry	Symmetry	Symmetry	Symmetry	N/A
**STEP VELOCITY TEST**						
CW Tc (sec)	4.58	1.22	–	4.67	2.37	N/A
CCW Tc (sec)	4.16	1.74	–	5.55	2.39	N/A
**VEMP (INITIAL)**						
Response (Rt/Lt)	(+/+)	(+/weak)	(+/+)	(+/+)	(+/+)	(+/+)
IAD (%)	5.8	52.1	12.1	4.6	18.3	36.5
**VEMP (LAST F/U)**						
Response (Rt/Lt)	(weak/+)	(–/–)	N/A	(weak/+)	(–/–)	N/A
IAD (%)	47.3	–	N/A	47.8	–	N/A
**VIDEO HIT**						
LHC gain	N/A	N/A	N/A	N/A	0.25	1.08
RHC gain	N/A	N/A	N/A	N/A	0.27	1.21
LAC gain	N/A	N/A	N/A	N/A	0.15	0.82
RAC gain	N/A	N/A	N/A	N/A	0.15	1.02
LPC gain	N/A	N/A	N/A	N/A	0.13	0.84
RPC gain	N/A	N/A	N/A	N/A	0.24	0.89

SPV, slow-phase velocity; RW, right warm; RC, right cold; LW, left warm; LC, left cold; F/U, follow-up; CW Tc, clockwise time constant; CCW Tc, counter-clockwise time constant; VEMP, vestibular-evoked myogenic potentials; HIT, head impulse test; LHC, left horizontal canal; RHC, right horizontal canal; LAC, left anterior canal; RAC, right anterior canal; LPC, left posterior canal; RPC, right posterior canal; N/A, not available; NR, no response

**Table 4:** The patterns of vestibulopathy, presence and characteristics of hearing impairment, and radiologic assessment.

**Subject**	**Patterns of vestibulopathy (initial)**	**Patterns of vestibulopathy (F/U)**	**Hearing impairment**		**MRI finding (hemosiderosis deposition)**		
			**SNHL^*†*^ (R/L)**	**OAE**	**Cerebellum**	**Brainstem**	**CN VIII**
1	Central	B) combined	M/M	N/A	Yes	Yes	Yes
2	B) peripheral	B) peripheral	P/P	NR	Yes	Yes	Yes
3	B) combined	B) combined	MS/P	N/A	Yes	Yes	Yes
4	B) combined	B) combined	P/P	NR	Yes	Yes	Yes
5	B) combined	B) combined	P/P	N/A	Yes	Yes	unclear
6	Central	Central	m/P	NR	Yes	No	No

† *We described final follow-up status based on pure tone audiogram. B, bilateral; R, right; L, left; F/U, follow-up; CN VIII, vestibulocochlear nerve; m, mild; M, moderate; MS, moderate to severe; P, profound; N/A, not available; NR, no response*.

### Subject 1 (F/78)

**Figure 1:**
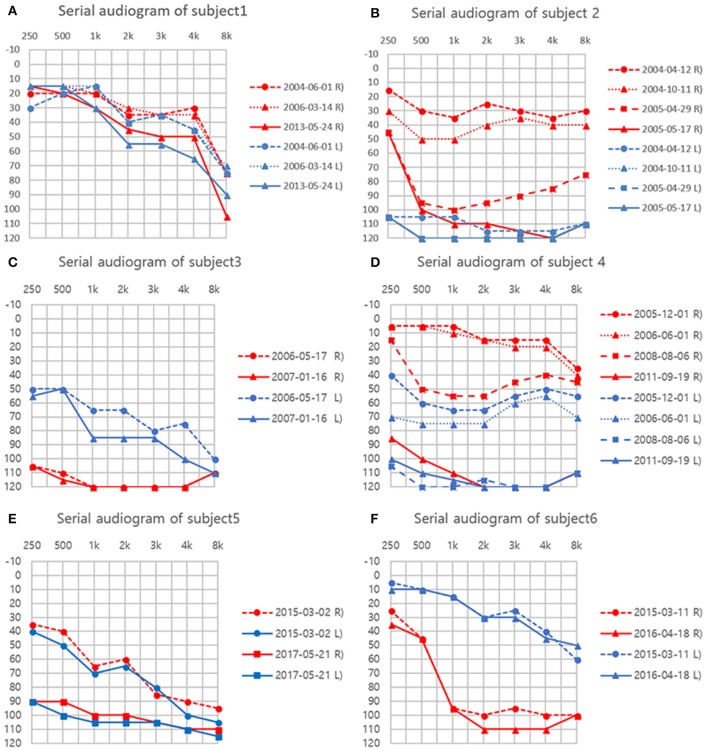
**(A–F)** Serial audiograms in individual patients. Air conduction thresholds (dB HL) at each frequency (Hz) are plotted for both ears.

**Figure 2:**
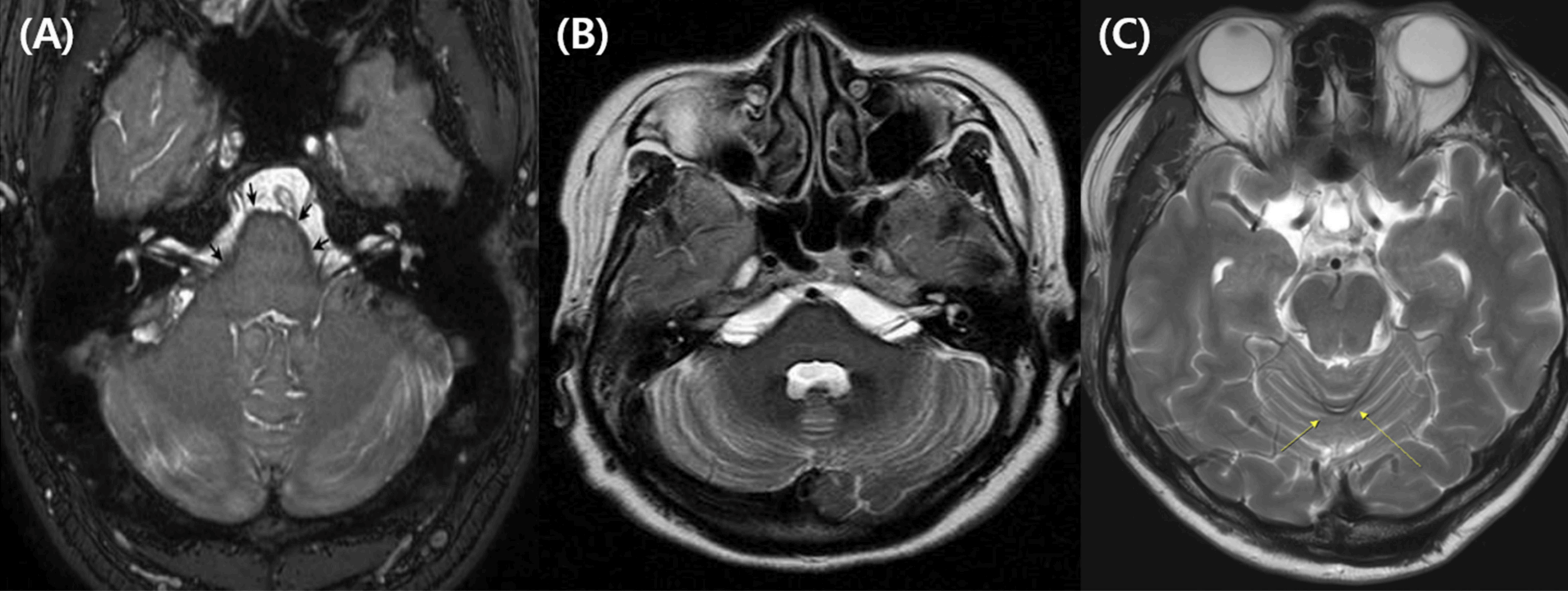
Representative caloric test results obtained during the follow-ups in subject 4. The responses to bithermal caloric irrigation were normal in both ears at initial evaluation. **(A)** But, deteriorated asymmetrically over the years. **(B)** Finally, bilateral caloric paresis became evident at the most recent examination **(C)**.

A 78-year-old female patient presented with cerebellar ataxia without hearing loss 11 years prior to her initial neurotological evaluation. Four years later, symmetrical mild hearing loss in both ears was observed on PTA. Speech discrimination (SD) also showed 92% on both ears. However, her hearing did not deteriorate further during follow-up PTA (Figure 1A). On VNG examination, spontaneous down-beating nystagmus (DBN) and right-beating nystagmus were documented, the intensities of which increased upon head-shaking, reflecting perverted DBN. Also, GEN, characterized by DBN augmentation during lateral- and up-gazing, was evident during the most recent examination. The ocular motor test revealed hypometric saccades with low-pursuit gain. The bithermal caloric test result was normal at initial evaluation; however, the test results deteriorated bilaterally during follow-up. During her most recent evaluation, the rotator chair test was compatible with BVH. Similarly, cVEMP test showed normal symmetric response on first examination; however, IAD (the right value was 47.3% that of the left) suggestive of right-sided saccular dysfunction were evident at the 2-year follow-up evaluation. She began to experience oscillopsia recently. T2-weighted and GRE images revealed superficial siderosis around the cerebellum and brainstem, additionally, both CN VIII were shown based on bTFE images of internal auditory canals (Figure 2A).

### Subject 2 (M/38)

Fourteen years ago, this patient suffered severe head trauma while engaging in whitewater rafting. Two years thereafter, he developed sudden hearing loss in the left ear and disequilibrium. Subsequently, he started to have hyposmia 3 years after trauma. Initial PTA revealed total deafness in the left ear and mild SNHL of 30 dB HLin the right ear. Hearing of the right ear also deteriorated to profound deafness over the following year (Figure 1B). Cochlear implantation was eventually performed. Postoperative open-set speech perception improved compared with the preoperative results: sentence test, 76%; mono-syllabic word test, 60%; bi-syllabic word test, 50%. On an exhaustive VNG examination, no definite nystagmus was documented. In addition, the ocular motor test revealed normal saccade amplitude and latency. Bithermal caloric tests revealed bilateral canal paresis, and the direction of nystagmus on supine and prone position was not changed during the ice-water test. Notably, VEMP elicited responses from both ears but the left was 52.1% smaller than that of the right on amplitude. At the 6-year follow-up, cVEMP response was not evident on either side. T2-weighted and GRE images revealed superficial siderosis around the cerebellum and brainstem, additionally, both CN VIII were shown based on bTFE images of internal auditory canal.

### Subject 3 (F/42)

This patient was diagnosed with a cervical spine chordoma 16 years ago and underwent several operations for tumor resection over the next 3 years. Seven years after her surgeries concluded, she presented with cerebellar ataxia. Over the following 3 years, she had developed bilateral hearing loss. She subsequently reported dysesthesia after bilateral hearing loss. The initial PTA revealed asymmetric SNHL (deafness and 0% SD in the right ear and 65 dB HL threshold and 4% SD in the left ear, Figure 1C). During an exhaustive VNG examination, subtle spontaneous DBN, and GEN, characterized by DBN with ipsilateral horizontal nystagmus during lateral gaze, were documented. The ocular motor test revealed a hypermetric saccade with a low pursuit gain. The bithermal caloric test revealed bilateral caloric paresis at initial evaluation, and the direction of nystagmus did not change during the ice-water test. The cVEMP responses were normal and the IAD was within the normal range on initial evaluationT2-weighted and GRE images revealed SS in the lining of the cerebellum, brainstem, and both CN VIIIs probably suspected by severe hemosiderin deposition surrounding internal auditory canal (Figure 2B).

### Subject 4 (F/52)

**Figure 3:**
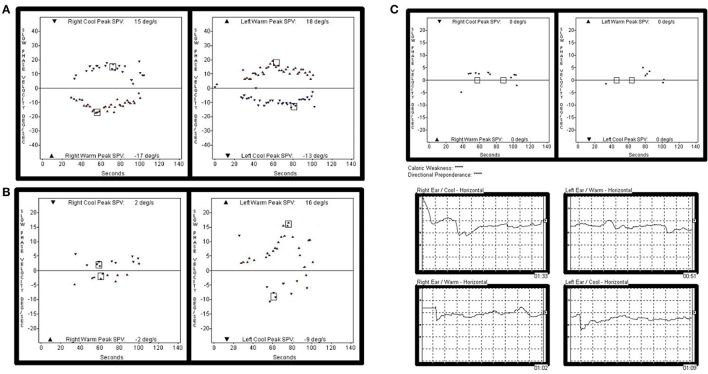
Representative MRIs illustrating hemosiderin deposition. **(A)** Balanced turbo field-echo (bTFE) image shows hemosiderin deposition lining the cerebellum, brainstem, and both vestibulocochlear nerves (subject 2, The arrow indicates the surface of the pons.). **(B)** T2-weighted image shows hemosiderin deposition around the cerebellum, brainstem, and both vestibulocochlear nerves (subject 3). **(C)** T2-weighted image shows hemosiderin deposition on the posterior cerebellum (subject 6).

This patient had a history of brain hemorrhage caused by a cavernous hemangioma in the right temporal lobe. Left side hemiparesis developed after a decompressive craniotomy. Eight years later, she initially presented with left-side hearing loss. One year later, she complained of cerebellar ataxia and disequilibrium. Initial PTA revealed unilateral SNHL (Rt:10dB HL and 100% SD, Lt: 60 dB HL and 36% SD). She then developed bilateral deafness over the following 5 years (Figure 1D). Cochlear implantation was eventually performed. Postoperative open-set speech perception improved compared with the preoperative results: sentence test, 70%; mono-syllabic word test, 60%; multi-syllabic word test, 50%. No specific nystagmus was noted on SN, GEN, HSN, and VIN test. The ocular motor test revealed bilaterally decreased pursuit gain without saccades. Both ears responded normally to bithermal caloric irrigation; however, right caloric paresis developed over the years and bilateral caloric paresis was evident at the most recent examination (Figure 3), at which time the rotator chair test indicated a phase lead, a decreased gain, but no definite asymmetry, compatible with BVH. Similarly, initial cVEMP indicated that the amplitude and latency of both ears were normal on initial evaluation; however, at the 3-year follow-up, the right-side IAD was 47.8% that of the left side. T2-weighted and GRE images revealed diffuse hemosiderin depositions around the cerebellum, brainstem, midbrain, and both CN VIII but the cavernous hemangioma exhibited no interval change over the years.

### Subject 5 (F/53)

**Figure 4:**
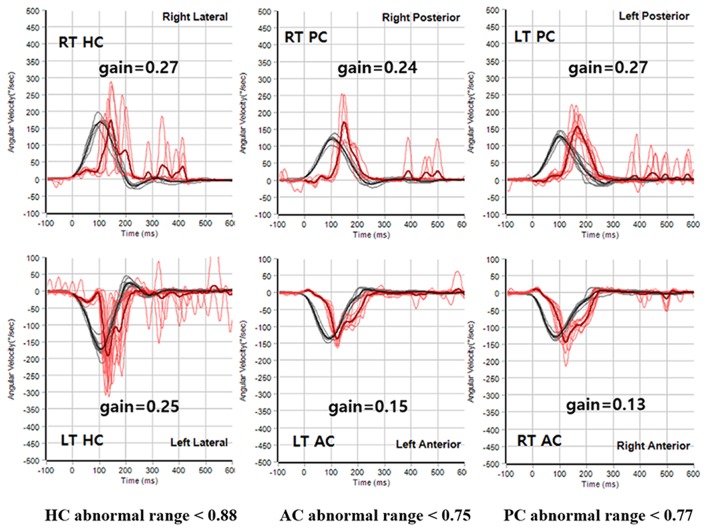
Representative video HIT results obtained during the follow-ups in subject 5. The VOR gains were reduced for all six semicircular canals during the recording of video HIT.

This patient had a history of spinal cord bleeding 6 years prior to her first visit. Two years later, she began to complain of cerebellar ataxia and bilateral hearing loss. Initial PTA revealed symmetrical SNHL (threshold: 60 dB HL, SD: 16%). Profound bilateral SNHL developed over the next 2 years (Figure 1E). She recently began to suffer from intermittent diplopia. On VNG examination, SN was absent, but GEN was evident during lateral gaze. The ocular motor test revealed hypsometric saccades and a low pursuit gain. The bithermal caloric test revealed bilateral caloric paresis, and no change in the direction of nystagmus was evident during the ice-water test. Moreover, video HIT revealed both overt and covert saccadic movements, and the VOR gains of all six semicircular canals were reduced (Figure 4). Notably, the cVEMP test was normal at initial evaluation, but was absent at the 2-year follow-up. T2-weighted and GRE images revealed diffuse hemosiderin deposition in the brain, particularly the cerebellum and brainstem. Additionally, T-spine MRI showed that the entire spinal cord exhibited SS.

### Subject 6 (M/65)

This patient was diagnosed with a cystic lumbar tumor 20 years ago and underwent several surgeries for tumor resection. He presented with cerebellar ataxia 10 years after the last operation. Seven years later, he developed right-side hearing loss. PTA revealed asymmetric SNHL (80 dB HL and 12% SD on the right, 30 dB HL and 100% SD on the left side; Figure 1F). During exhaustive VNG examination, subtle spontaneous DBN with left-beating nystagmus was documented. The ocular motor test indicated a hypermetric saccade without a pathological pursuit gain. At initial evaluation, his responses to bithermal caloric irrigation were within the normal range, and video HIT revealed normal VOR gains in all six semicircular canals. In addition, the cVEMP test demonstrated that the hearing thresholds and latencies were normal There were no additional abnormal neurologic findings. T2-weighted and GRE images revealed SS only in the superior cerebellum (Figure 2C).

### Patterns of vestibular presentation

We list the patterns of vestibulopathy found during follow-up in Table 4. When hearing impairment was evident on the most recent vestibular work-up, bilateral combined central and peripheral vestibulopathy was the most common vestibular presentation; four of the six cases (subjects 1, 3, 4, and 5) presented with cerebellar ataxia, neuropathy, and vestibular areflexia syndrome(CANVAS) due to combined central and peripheral vestibulopathy on both sides. Of the remaining two patients, however, one exhibited bilateral vestibulopathy without central signs or cerebellar ataxia, and the other showed cerebellar ataxia and central signs without peripheral vestibular dysfunction, consistent with isolated central vestibulopathy.

Of note, the patterns of vestibulopathy had evolved in two patients during the follow-up (subjects 1 and 4). For example, the subject 1 with spontaneous DBN, GEN and progressive cerebellar ataxia, but normal caloric test and cVEMP initially showed an isolated central vestibulopathy, but later conversion into bilateral combined central and peripheral vestibulopathy.

### Radiological manifestations

Radiologically, hypo-intense lesions surrounding the cerebellum and brainstem were evident on T2-weighted and GRE MRIs of all patients, but one patient lacked such lesions around the brainstem. The hypo-intense lesions were visualized along both CN VIII on bTFE images of the internal auditory canal in two patients and were suspected on T2-weighted and GRE images in another two patients. Although cochleo-vestibular dysfunction usually progressed asymmetrically, asymmetry of the MRI lesions was not evident. Table 4 summarize the radiological extents and locations of hemosiderin deposits.

## Discussion

All patients exhibited SNHL, which progressed over the years in five of the six patients. Similarly, heterogeneous vestibular patterns were observed during the disease process, but most patients exhibited combined bilateral peripheral and central vestibulopathy at their most recent evaluations. Also, although cochlea-vestibular dysfunction was mostly bilateral and progressed asymmetrically, no asymmetry of hemosiderin deposition was evident on MRIs. Thus, precise identification of cochlea-vestibular dysfunction and central signs is essential in SS, and patients with SS should undergo regular, comprehensive neurotological evaluation to optimize their treatments and prognosis.

### Vestibular characteristics and the clinical course of superficial siderosis

SS is associated with slow progressive deterioration (5), and vestibular status can vary over time. However, SS patients ultimately suffer from functional decline of either the central or peripheral vestibular system. Recently, a correlation between central ataxia and bilateral vestibulopathy has been noted (33). Also, recent reports have suggested that when chronic bilateral combined vestibulopathy is associated with hearing impairments, SS may be the most common cause (7, 9). In the present study, four of six patients exhibited bilateral combined central and peripheral vestibulopathy on their most recent evaluations. The other two patients exhibited bilateral peripheral vestibulopathy or isolated central vestibulopathy.

Several studies have reported various patterns of vestibulopathy, however, all were cross-sectional in nature (3, 5–9, 11–22). The median follow-up of our cases was 12 years (range, 3–15years). In this study, we showed that vestibulopathy is heterogeneous during follow-up. Notably, of four patients with bilateral combined vestibulopathy, evolution of the vestibular pattern from isolated central vestibulopathy into bilateral combined central and peripheral vestibulopathy was evident in two patients. The caloric function and cVEMP test results were normal at the initial evaluation, and then deteriorated asymmetrically during follow-up. However, the comparison of each side would be meaningless in markedly decreased response. In patients with chronic bilateral combined vestibulopathy, conspicuous cerebellar dysfunction may mask peripheral vestibular involvements (9). A previous study suggested that such patients would find it challenging to develop central adaptation for their imbalance because bilateral vestibulopathy weakens primary vestibular function (33). Thus, the progressive and bilateral nature of the pathology is important when planning treatment and predicting prognosis. Identification of the precise vestibular status and central signs via regular, comprehensive neurotological evaluation may be the key for optimization of both treatment and prognosis (9).

Moreover, all patients exhibited SNHL (mostly asymmetric and progressive), in line with the findings of a previous longitudinal study on the audiological characteristics of SS (6). Typically, SS presents bilateral, asymmetrically progressive, cochlea-vestibular dysfunction combined with cerebellar ataxia. However, although cochlea-vestibular dysfunction usually progresses asymmetrically, no asymmetry of hemosiderin deposition was evident on MRIs in the present study. Also, previous studies reported that the extent and distribution of deposits evident on MRI did not necessarily correlate with the severity of clinical manifestations (34, 35). Although this is an enigmatic finding, it has recently been suggested that certain physiological pathways protecting the CNS against intracranial iron overload may become activated in SS patients (36).

### Specific vestibular signs in superficial siderosis

Neurotologically, DBN may constitute diagnostic vestibular evidence of SS. In the present study, DBN developing either spontaneously or after head-shaking was evident in three of the six patients. Although the mechanism of DBN remains unclear, asymmetry of the cerebellum-brainstem network and an imbalance between the downward and upward vestibular tracts, including the superior vestibular nucleus-ventral tegmental tract, may generate DBN (37, 38). Hemosiderin deposits in the cerebellum or brainstem were common radiological characteristics of our patients. Selective hemosiderin deposition in the cerebellum interferes with the cerebellum-brainstem network and compromises vertical vestibular-cerebellum neural integration, causing DBN. In addition, hemosiderin deposition on the brainstem, which lies on the course of the superior vestibular nucleus-ventral tegmental tract (39), may compromise the normal functioning of that tract, inducing vertical nystagmus. Recently, a relationship between CANVAS and DBN was found in a large series of patients with DBN, which was associated with additional signs including bilateral vestibulopathy, cerebellar ataxia, and peripheral neuropathy (40, 41). The vestibular test batteries used in the present study showed that cerebellar ataxia was present in all three patients with DBN and bilateral vestibulopathy in two of them. Furthermore, bilaterally positive HIT, impaired pursuit gains, and GEN were documented in more than half of the patients. Similarly, a previous study on CANVAS patients suggested that impairment of the visually enhanced VOR was a typical sign of combined vestibulopathy because both smooth pursuit and the VOR were simultaneously affected in such patients (42).

The cVEMP test is regarded reliable when used to evaluate the integrity of the saccule, inferior vestibular nerve, and its central connections (43). All patients of the present study yielded normal cVEMP test results at their initial evaluations, supporting the findings of previous studies having shown that otolithic function is preserved in SS patients (3, 5). The cVEMP has contributions from different areas of the cerebellum. Hemosiderin deposition may initially target the cerebellar flocculus, which mostly modulate the angular VOR control, but not the cerebellar nodulus that is more closely related to the otolith reflexes (44). Furthermore, as hemosiderin deposition in CNS persists for at least several months (45), cVEMP responses tend to be impaired in patients who have suffered from SS for longer periods, and not in those with early-stage disease. The four patients who underwent follow-up cVEMP developed significant IAD or lost their cVEMP responses over the years.

Although both caloric function and the cVEMP deteriorated during follow-up, considerable differences were evident on initial evaluation. The caloric test, which evaluates the VOR (46), indicated vestibulopathy in four of the six patients, but cVEMPs were all normal. Thus, the superior vestibular nerve appears to be more affected by hemosiderin deposition than the inferior vestibular nerve. Anatomically, the superior vestibular nerve is longer than the inferior vestibular nerve, and traverses small, osseous neural canals (47). In addition, more of the surface area of the superior vestibular nerve is in contact with the cerebrospinal fluid (CSF). Thus, the superior vestibular nerve has a greater glial segment susceptible to iron impregnation (47).

### Vestibular pathophysiology in superficial siderosis

The clinical manifestations of SS depend on the sites and extents of hemosiderin deposition (1). We found that the cerebellum and brainstem were the most commonly affected regions, consistent with the previous studies. The cerebellum has a large, folded surface, which may make it susceptible to iron deposition. These sites are exposed to high levels of CSF (1, 4). Chronic bleeding into the subarachnoid space increases the CSF hemoglobin level, and heme oxygenase produced by glial or microglial cells cleaves free heme into biliverdin and iron. Thus, iron deposits in this region are common in patients exhibiting gliosis, neuronal loss, and demyelination (48). Also, iron deposition increases hydroxyl radical production, causing oxidative stress and tissue damage (49). Thus, oxidative cellular damage, accompanied by reactive gliosis, neuronal loss, and demyelination associated with hemosiderin deposition may weaken the pathways that must be active to counter any decline in VOR gain (50). Such a pathological cascade could cause the characteristic progressive cerebellar ataxia and progressive SNHL evident in the present study (51). Our findings are similar to those of a previous study; progressive SNHL and progressive cerebellar ataxia developed in 95 and 88% of cases, respectively.

In addition, hemosiderin can be deposited along the cranial nerves. In particular, the CN VIII running through the pontine cistern has a long glial segment and is exposed to high-level CSF flow, rendering the nerve particularly susceptible to iron deposition (52). Also, the inner ear structures can also be affected in SS (12). In support of these findings, an earlier histopathological study of the temporal bone showed that atrophy of CN VIII, the loss of hair cells (53), and subsequent fibrosis, contributed to impairment of peripheral blood flow in the inner ear (12). Moreover, a previous report suggested that chronic hemorrhage directly affected the inner ear structures, precipitating neurotological symptoms (54). Likewise, loss of smell sensation may be an initial feature of SS since the olfactory tract and bulb can also be affected during the earlier phase of this disorder (55).

### Limitations and future perspectives

To the best of our knowledge, this is the first study to discuss vestibular manifestations over time in a relatively large SS cohort. Although our data will be useful in terms of diagnostic evaluation and will assist future clinical and basic studies on SS, some limitations of our work remain to be addressed. First, because our sample was relatively small, we cannot conclude that we encountered all possible vestibular manifestations; a prospective larger cohort study is necessary. Second, given the heterogeneity in vestibular function evident among the studies, it is difficult to describe associations between vestibular function and SS; a unified protocol for vestibular evaluation is required. Third, although the location and extent of hemosiderin deposition revealed by MRI can plausibly be used to explain the vestibular pathophysiology, the disease is multifactorial in nature; temporal bone histopathological data and more accurate neuroimages would be helpful. Lastly, we generally used caloric paresis as a marker of peripheral vestibular involvement, which is a well-known feature of SS. However, caloric paresis may reflect brainstem pathology involving the vestibular fascicle or the nuclei (56).

## Conclusion

SS typically presents bilaterally asymmetric, progressive audiovestibular dysfunction with cerebellar ataxia. The most common pattern of vestibular dysfunction is bilateral combined central and peripheral vestibulopathy. Thus, precise identification of audiovestibular dysfunction and central signs is essential in SS, and patients with SS should undergo regular, comprehensive neurotological evaluation to optimize their treatments and prognosis.

## Author contributions

S-YL designed and performed experiments, analyzed data and wrote the paper; J-WK conceived the study and wrote the paper; D-HL, YB, and J-JS collected and analyzed data; J-WK, and JK revised the article critically for important intellectual content. All authors discussed the results and implications and commented on the manuscript at all stages.

### Conflict of interest statement

The authors declare that the research was conducted in the absence of any commercial or financial relationships that could be construed as a potential conflict of interest.
